# The G allele of the *IGF1* rs2162679 SNP is a potential protective factor for any myopia: Updated systematic review and meta-analysis

**DOI:** 10.1371/journal.pone.0271809

**Published:** 2022-07-21

**Authors:** Bo Meng, Kang Wang, Yingxiang Huang, Yanling Wang

**Affiliations:** Beijing Friendship Hospital, Capital Medical University, Beijing, China; National Eye Institute, UNITED STATES

## Abstract

**Background:**

The insulin-like growth factor 1 (*IGF1*) gene is located within the myopia-associated MYP3 interval, which suggests it may play an important role in the progression of myopia. However, the association between *IGF1* SNPs and any myopia is rarely reported.

**Methods:**

A comprehensive literature search was conducted on studies published up to July 22, 2021 in PubMed, EMBASE, CBM, COCHRANE, CNKI, WANFANG and VIP databases. Odds ratios (ORs) and 95% confidence intervals (CIs) were calculated for single-nucleotide polymorphisms (SNPs) that have been evaluated in at least three studies.

**Results:**

Nine studies involving 4596 subjects with any myopia and 4950 controls examined 25 SNPs in *IGF1* gene, among which seven SNPs were included in this meta-analysis. Significant associations were not found in any genetic models between rs6214, rs12423791, rs5742632, rs10860862, rs5742629 and any myopia. Rs2162679 was suggestively associated with any myopia in the codominant model (GA vs. AA: OR = 0.87, 95% CI: 0.76–1.00) and the dominant model (GG+GA vs. AA: OR = 0.88, 95% CI = 0.78–1.00).

**Conclusion:**

Meta-analysis of updated data reveals that the G allele of the *IGF1* rs2162679 SNP is a potential protective factor for any myopia, which is worth further researches.

## Introduction

Recently, myopia has emerged as a major public health concern worldwide. In the last several decades, the prevalence of myopia in the United States and Europe has increased [1, 2]. Asian countries have the highest rates of myopia, especially in east and Southeast Asia [3]. In China, Singapore and Taiwan, the prevalence of myopic subjects aged 12–39 years has rapidly increased to 67–96% [4–6]. Because of its higher prevalence, myopia imposes enormous economic and social burdens worldwide [7].

Although myopia is classified as a benign disorder that can be corrected with optical modalities, myopic eyes with a long axial lengths (≥26 mm) or a high degree of myopic refractive error (≤−6D), can cause blindness with complications such as glaucoma, macular degeneration, retinal detachment, myopic foveoschisis, and choroidal neovascularization [8, 9]. Myopia has already become the second most common cause of legal blindness [10, 11]. Therefore, it is very important to identify the potential risk factors to establish preventive strategies for myopia.

The pathogenesis of myopia remains unclear. Research has shown that myopia is a multi-factorial disease that results from an interaction between environmental and genetic factors [12–14]. Environmental factors include near work, outdoor activities, level of education, light exposure, diet and urbanization [15, 16]. For example, in two independent population-based cohorts of individuals from European descent, Verhoeven et al. [17] found that the genetic risk of an individual for myopia is significantly affected by his or her educational level. Higher education affects myopia by increasing the amount of time spent doing near work activities [18]. By contrast, children who spend more time engaged in outdoor activities have shown a reduced prevalence and a slower progression of myopia. Although the environment plays a role in the progression of myopia, results of twins and family-based studies have shown that the genetic component is significant [19, 20]. Association studies have led to the identification of many susceptibility and causative genes for myopia. These genes are enriched for certain functional annotations, such as neurotransmitter functions (GRIA4), ion channel activity (KCNQ5, CD55 and CACNA1D), retinoic acid metabolism (RDH5, CYP26A1 and RORB), extracellular matrix remodeling (LAMA2 and BMP2) and ocular development (SIX4, CHD7 and PRSS56) [21].

The *IGF1* gene is located in 12q23.2 of the human genome and contains six exons [22]. One of the proteins encoded by this gene is similar to insulin in its structure and function. Previous animal studies showed that the *IGF1* gene contributed to eye development and disease. For example, *IGF1*/*FGF2*-treated eyes in animal studies could have an increased vitreous chamber depth, decreased anterior chamber depth, and changes in the sclera [23]. Hellstrom et al. showed that lack of *IGF1* in knockout mice prevented normal retinal vascular growth by preventing VEGF-induced activation of protein kinase B, a kinase that is critical for endothelial cell survival [24]. Additionally, Ruberte et al. [25] suggested that *IGF1* played a role in the development of ocular complications in patients with diabetes for a long period of time. The *IGF1* gene also is located within the myopia-associated MYP3 interval, which has been mapped using the linkage disequilibrium method. This suggests that *IGF1* may play an important role in the progression of myopia. However, the association between *IGF1* SNPs and any myopia is rarely reported. Therefore, we present herein an updated systematic review and meta-analysis to evaluate the potential association between *IGF1* SNPs and any myopia.

## Methods

### Search strategy

The review protocol was registered with the International Prospective Register of Systematic Reviews (PROSPERO, CRD42021274322) and performed according to the Preferred Reporting Items for Systematic review and Meta-Analyse Statement (PRISMA) guidelines. We searched the following databases: PubMed, EMBASE, Cochrane Library and several Chinese databases, such as the Chinese biomedical literature database (CBM), China National Knowledge Infrastructure (CNKI), WANFANG DATA and VIP database from their inception to July 22, 2021. The selected key words were used as free words, truncations and MeSH terms. Reference lists from the retrieved articles were manually screened for potential articles, if any, that had not been captured by the electronic search. No language restrictions were applied throughout the search process.

### Inclusion and exclusion criteria

Inclusion criteria were as follows: 1) original case-control or family-based studies that evaluated the association between polymorphisms of *IGF1* and any myopia; 2) numbers or frequencies in case and control groups reported for each genotype or allele; 3) if the study was reported in duplicate, the version with the most comprehensive content was included; and 4) studies including normal individuals with spherical equivalent refraction that ranged from -1.5 to 1.5 diopters and were free from any complications.

Exclusion criteria were as follows: 1) animal studies, reviews, conference proceedings, case reports, editorials; and 2) articles providing incomplete data or that could not be acquired through various means.

### Data extraction

Two independent authors screened all retrieved records and made decisions on which studies to include. Any disagreements were resolved by discussion. Further, any uncertainties were resolved by consultation with a third author. The information of first author, year of publication, ethnicity, genotyping type, sample size, polymorphisms studied, genotype distribution, minor allele, Hardy–Weinberg equilibrium (HWE) and conclusions on any myopia association were collected. If allele data were not available in original reports, they were calculated based on genotypic data.

### Assessment of study quality

Study quality was assessed using revised criteria according to Little’s recommendations [26] for gene-disease associations, with an aim to investigate potential bias in summary results. These criteria included: 1) the genotyping method used; 2) definition of cases and methods of ascertainment; 3) socio-demographic characteristics of subjects; 4) confounding factors mentioned in articles; and 5) confidence intervals of genotype frequency. An overall quality score was generated, and studies with a score ≥3 were considered to have high quality.

### Statistical analysis

All statistical analyses were performed using RevMan 5.3. Association of each SNP with myopia in pooled samples, along with pooled odds ratios (ORs) and 95% confidence intervals (95% CIs), were evaluated. The I^2^ statistic was used to quantify heterogeneity. In addition, funnel plot was used to evaluate the publication bias.

## Results

### Eligible studies and study characteristics

**Fig 1:**
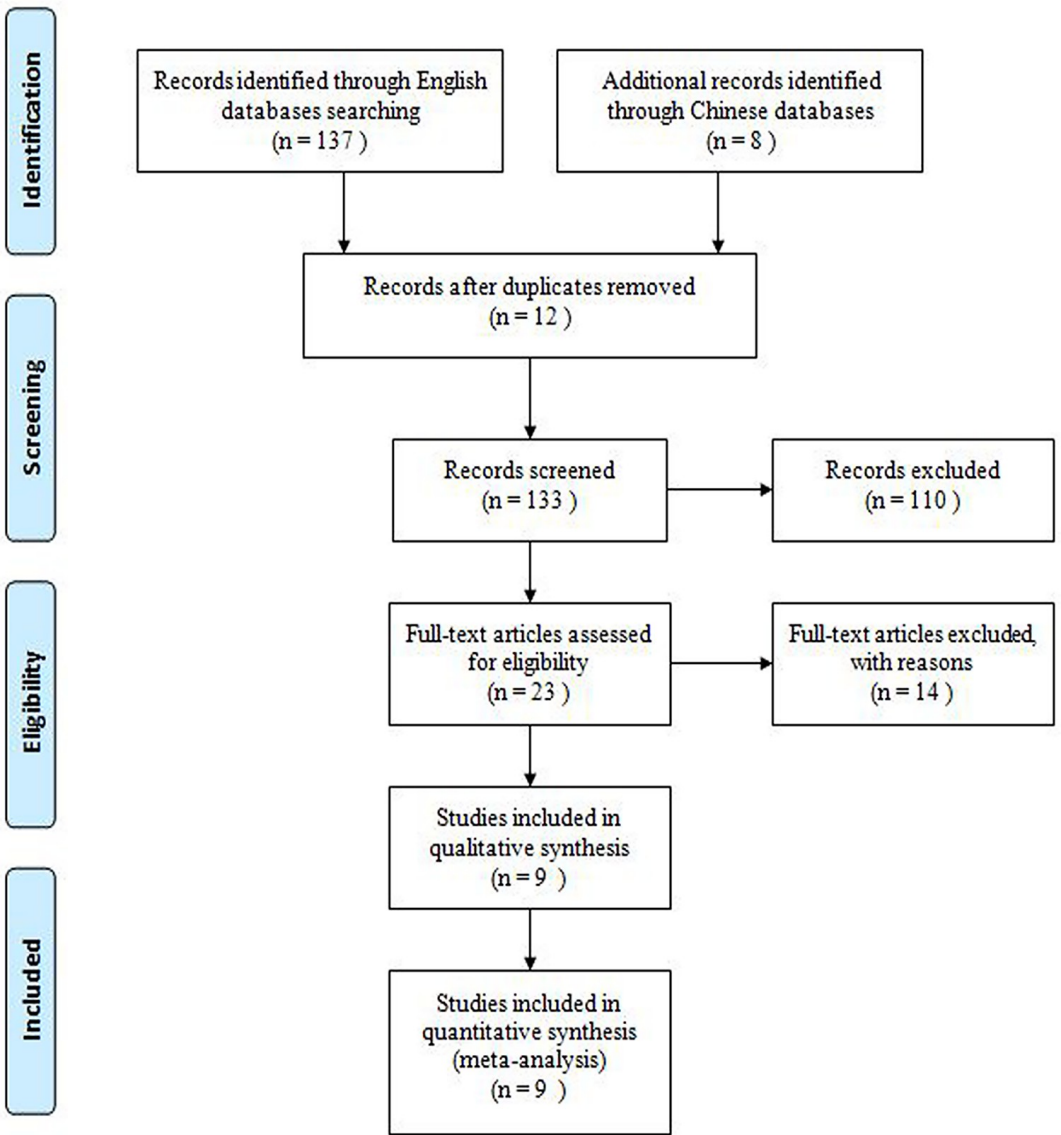
Flowchart of study inclusion.

A total of 145 potentially relevant articles were retrieved. Ultimately, nine studies that met all criteria were included for this meta-analysis (Fig 1) [27–35].

Overall, 25 SNPs associated with the *IGF1* gene were investigated at least once in nine studies. Among these SNPs, seven were tested in at least three studies and then were included in the meta-analysis. The study subjects were Chinese [29, 31, 32, 34, 35], Japanese [27, 28], Egyptian [33] and Polish [30] with sample sizes that ranged from 127 to 1339. The total sample size was 9546 (4596 individuals with any myopia and 4950 controls).

The methods of gene analysis included restriction fragment length polymorphism (RFLP), matrix-assisted laser desorption/ionization time-of-flight mass spectrometry (MALDI-TOF), RT-PCR, SnaPshot and polymerase chain reactionand ligase detection reaction (PCR-LDR). The quality scores of the included studies were greater than four, which indicated a favorable methodological quality. Table 1 summarizes the characteristics of the included studies.

**Table 1:** Characteristics of all studies included in the meta-analysis.

First author	Year	Ethnicity	Genotying type	Quality score	SNP ID	Sample		Mean age(y)		Mean refractive errors (D)		Genotype distribution						Minor allele	HWE
												Case			Control				
						Case	Control	Case	Control	Case	Control	1/1	1/2	2/2	1/1	1/2	2/2		
**Cheng**	2020	Chinese	PCR-LDR	5	rs6214	281	373	9.84±1.55	8.06±1.43	-2.55±1.64^▲^	0.84±0.81^▲^	59	140	82	89	186	98	A	yes
					rs5742653					-2.55±1.84^△^	0.88±0.83^△^	62	140	79	83	186	104	G	yes
					rs4764697							9	83	189	10	102	261	T	yes
					rs12423791							16	103	162	27	146	200	C	yes
					rs2162679							29	122	130	52	175	146	G	yes
					rs5742612							21	112	148	34	157	182	C	yes
**Zidan**	2016	Egyptian	RFLP	4	rs5742632	136	272	41.2±9.0	42.23±8.0	-4.41±1.42^▲^^★^;-9.34±3.1^▲^^☆^	0.57±0.32^▲^	27	97	148	11	45	80	C	N/A
					rs6214			40.7±8.7		-4.39±1.4^△^^★^;-9.28±2.9^△^^☆^	0.59±0.31^△^	44	123	105	12	46	78	A	N/A
**Wang**	2016	Chinese	SNaPshot	5	rs10860860	1244	1380	41.26±13.51	58.39±12.77	-10.12±3.45^▲^	N/A	31	331	882	36	373	971	T	no
					rs10860862					-10.03±3.16^△^		38	357	849	41	393	946	T	no
					rs2946834							221	606	417	252	675	453	T	yes
					rs6214							280	620	344	321	689	370	A	yes
					rs12821878							3	121	1120	5	163	1212	A	yes
					rs35766							130	525	586	186	596	598	G	yes
**Zhao**	2013	Chinese	TOFMS	5	rs10860861	302	401	1.24±16.34	43.32±22.15	-16.54±5.26^▲^	0.39±0.82^▲^	44	148	110	66	197	138	C	yes
					rs10860862					-16.39±5.47^△^	0.42±0.80^△^	8	84	210	12	117	272	T	yes
					rs6214							89	145	68	101	200	100	G	yes
					rs5742629							48	167	87	58	186	157	G	no
					rs12423791							26	127	149	24	136	241	C	yes
					rs35766							134	131	37	207	157	37	G	yes
					rs1457601							18	130	154	21	140	240	A	yes
**Miyake**	2013	Japanese	TaqMan	4	rs6214	1339	1194	57.2±14.9	50.3±15.9	-12.69±4.54^▲^	N/A	277	641	373	268	585	341	C	yes
					rs978458							256	661	361	264	596	334	T	yes
					rs5742632							209	657	410	229	586	379	G	yes
					rs12423791							97	452	672	85	468	641	C	yes
					rs2162679							178	540	569	149	541	504	C	yes
**Yoshida**	2013	Japanese	TaqMan	5	rs6214	446	481	37.9±11.9	39.3±11.0	-11.7±2.24^▲^	-1.5~+1.5	58	205	183	55	215	211	G	yes
					rs11111262					-11.7±2.27^△^		17	138	291	18	150	313	A	yes
					rs972936							93	221	132	118	240	123	G	yes
					rs5742629							70	214	162	94	237	150	G	yes
					rs12423791							32	174	240	45	204	232	C	yes
					rs2162679							44	193	209	55	215	211	G	yes
					rs5742612							41	188	217	50	211	220	C	yes
**Zhuang**	2012	Chinese	MALDI-TOF	5	rs10860861	421	401	38.29±16.57	68.77±10.65	-14.57±5.6^▲^	0.39±0.82^▲^	153	202	66	138	197	66	C	yes
					rs10860862					-14.51±5.64^△^	0.42±0.8^△^	294	117	10	272	117	12	T	yes
					rs6214							99	205	117	100	200	101	G	yes
					rs5742629							128	222	71	157	186	58	G	yes
					rs12423791							219	170	32	241	136	24	C	yes
					rs35766							44	187	190	37	157	207	G	yes
					rs1457601							217	180	24	240	140	21	A	yes
**Mak**	2012	Chinese	RFLP	5	rs12579077	300	300	18–45	18–45	≤-8.0	-1.0~+1.0	38	109	153	36	128	136	C	yes
					rs35767							46	126	128	47	134	119	T	yes
					rs4764698							30	115	155	28	128	144	C	yes
					rs12423791							29	132	139	30	135	135	G	yes
					rs7956547							5	83	212	5	74	221	G	yes
					rs5742632							62	150	88	58	153	89	C	yes
					rs2373721							6	80	203	7	80	213	G	yes
					rs6539035							5	78	217	6	71	223	G	yes
					rs6214							74	146	80	85	137	78	A	yes
					rs5742723							30	118	152	31	127	142	A	yes
**Rydzanicz**	2011	Polish	RFLP	4	rs6214	127	148	27.1±22.63	38.6±18.54	-2.75±2.00^▲^	-0.03±1.26	22	72	64	16	78	54	A	yes
					rs10860860			40.2±20.43		-9.32±3.89^△^		18	68	72	13	75	60	T	yes
					rs2946834							19	62	75	14	61	72	T	yes

HWE: Hardy-Weinberg Equilibrium; N/A: Not available; ^▲^Right eye

^△^Left eye

^★^Simple myopia

^☆^High-grade myopia; 1/1: genotype with homozygous allele 1; 1/2: genotype with heterozygous alleles; 2/2: genotype with homozygous allele 2.

### Association of IGF1 SNPs with any myopia

**Fig 2:**
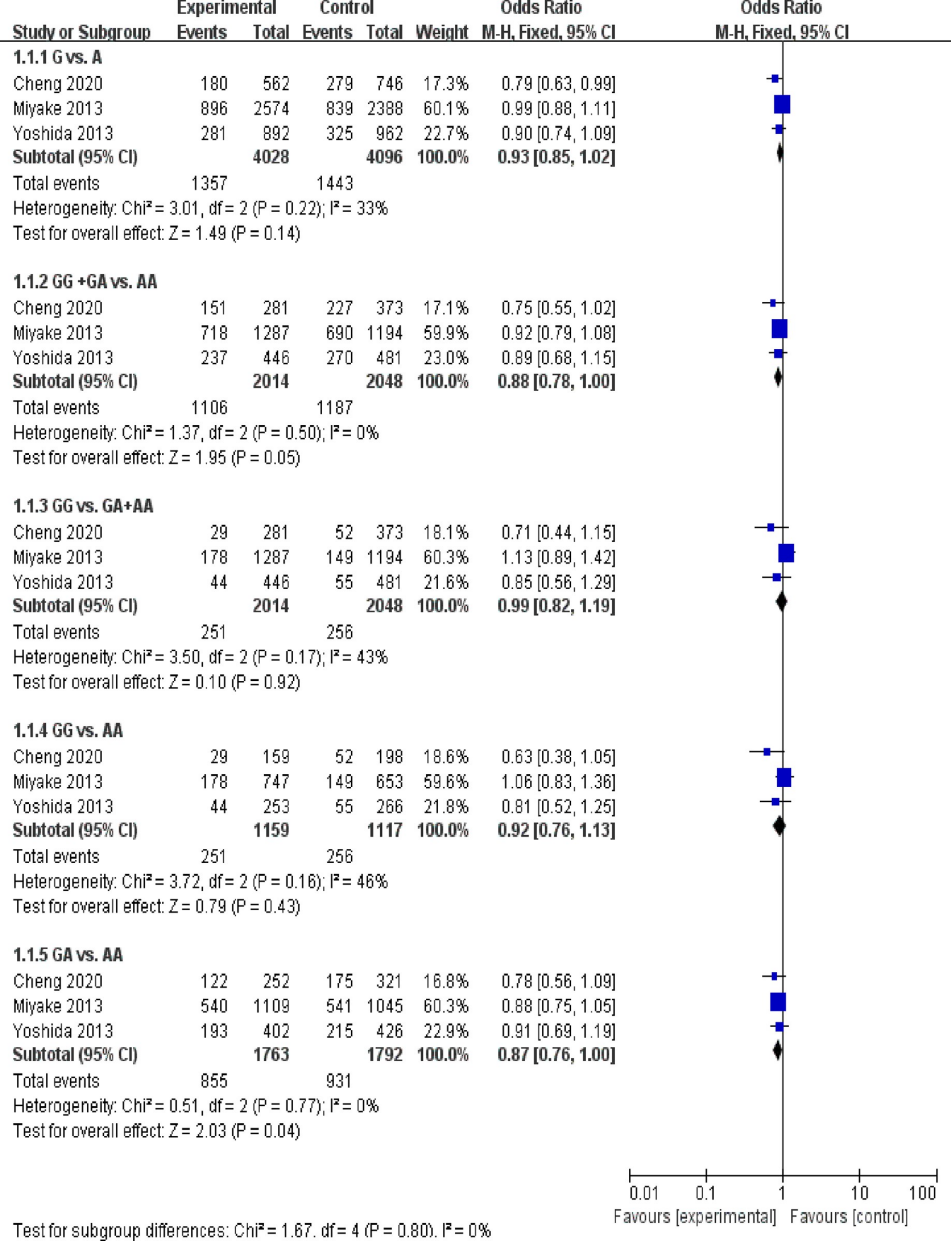
Meta-analysis of the association of *IGF1* rs2162679 with any myopia. Bars with squares in the middle represent 95% confidence intervals (95% CIs) and odds ratios (ORs). The central vertical solid line indicates ORs for the null hypothesis.

Rs2162679 was tested in three studies [27, 28, 32] with 2014 cases and 2048 controls. Fixed -effects models were used to calculate the pooled ORs. Our findings suggested that there were no significant associations for the allelic model (G vs. A: OR = 0.93, 95% CI: 0.85–1.02, *P* = 0.14), dominant model (GG+GA vs. AA: OR = 0.88, 95% CI = 0.78–1.00, *P* = 0.05), recessive model (GG vs. GA+AA: OR = 0.99, 95% CI = 0.82–1.19, *P* = 0.92 and codominant model (GG vs. AA: OR = 0.92, 95% CI = 0.76–1.13, *P* = 0.43). There were suggestive associations for the codominant model (GA vs. AA: OR = 0.87, 95% CI = 0.76–1.00, *P* = 0.04) (Fig 2, Table 2).

**Table 2:** Main results of the pooled ORs between IGF1 SNPs and any myopia.

SNPs	Models Tested		NO. study	Pooled OR	95% CI	*P*	*P*_*Q*_	I^2^
**rs2162679**	Allelic model	G vs. A	3	0.93	0.85–1.02	0.14	0.22	33%
	Dominant model	GG+GA vs. AA	3	0.88	0.78–1.00	0.05	0.5	0%
	Recessive model	GG vs. GA+AA	3	0.99	0.82–1.19	0.92	0.17	43%
	Codominant model	GG vs. AA	3	0.92	0.76–1.13	0.43	0.16	46%
		GA vs. AA	3	0.87	0.76–1.00	0.04	0.77	0%
**rs6214**	Allelic model	A vs. G	9	0.98	0.91–1.06	0.64	0.02	58%
	Dominant model	AA+AG vs. GG	9	1.03	0.90–1.18	0.65	0.04	50%
	Recessive model	AA vs. AG+GG	9	1	0.89–1.11	0.94	0.31	14%
	Codominant model	AA vs. GG	9	1.02	0.87–1.20	0.82	0.11	39%
		AG vs. GG	9	1.02	0.90–1.15	0.73	0.17	31%
**rs12423791**	Allelic model	C vs. G	6	0.95	0.81–1.11	0.51	0.005	70%
	Dominant model	CC+CG vs. GG	6	0.96	0.80–1.16	0.68	0.03	61%
	Recessive model	CC vs. CG+GG	6	0.92	0.73–1.15	0.45	0.14	40%
	Codominant model	CC vs. GG	6	0.93	0.71–1.22	0.61	0.13	41%
		CG vs. GG	6	0.97	0.82–1.16	0.76	0.09	48%
**rs5742632**	Allelic model	C vs. G	3	0.97	0.88–1.07	0.57	0.38	0%
	Dominant model	CC+CG vs. GG	3	1.01	0.88–1.17	0.88	0.69	0%
	Recessive model	CC vs. CG+GG	3	0.89	0.75–1.07	0.22	0.32	13%
	Codominant model	CC vs. GG	3	0.91	0.75–1.12	0.38	0.39	0%
		CG vs. GG	3	1.04	0.90–1.21	0.59	0.86	0%
**rs10860862**	Allelic model	T vs. G	3	1.02	0.91–1.14	0.8	0.7	0%
	Dominant model	TT+TG vs. GG	3	1	0.87–1.16	0.98	0.76	0%
	Recessive model	TT vs. TG+GG	3	1.06	0.84–1.35	0.62	0.89	0%
	Codominant model	TT vs. GG	3	1.05	0.73–1.51	0.81	0.81	0%
		TG vs. GG	3	1	0.86–1.16	1	0.83	0%
**rs35766**	Allelic model	G vs. A	3	0.93	0.74–1.16	0.51	0.01	78%
	Dominant model	GG+GA vs. AA	3	0.95	0.69–1.31	0.77	0.02	74%
	Recessive model	GG vs. GA+AA	3	0.81	0.65–1.00	0.05	0.24	29%
	Codominant model	GG vs. AA	3	0.83	0.56–1.21	0.32	0.07	62%
		GA vs. AA	3	1.01	0.77–1.32	0.97	0.08	60%
**rs5742629**	Allelic model	G vs. A	3	0.94	0.71–1.25	0.67	0.002	84%
	Dominant model	GG+GA vs. AA	3	1.02	0.65–1.59	0.94	0.003	83%
	Recessive model	GG vs. GA+AA	3	0.81	0.62–1.06	0.13	0.15	47%
	Codominant model	GG vs. AA	3	0.87	0.53–1.42	0.58	0.02	75%
		GA vs. AA	3	1.09	0.73–1.65	0.67	0.01	78%

Rs6214 was tested in nine studies [27–29, 31–36] with 4715 cases and 4814 controls. Random -effects models were used to calculate the pooled ORs. Our findings suggested that there were no significant associations for the allelic model (A vs. G: OR = 0.98, 95% CI: 0.91–1.06, *P* = 0.64), dominant model (AA+AG vs. GG: OR = 1.03, 95% CI = 0.90–1.18, *P* = 0.65), recessive model (AA vs. AG+GG: OR = 1.00, 95% CI = 0.89–1.11, *P* = 0.94 and codominant model (AA vs. GG: OR = 1.02, 95% CI = 0.87–1.20, *P* = 0.82 and AG vs. GG: OR = 1.02, 95% CI = 0.90–1.15, *P* = 0.73) (Fig a in S1 File, Table 2).

Rs12423791 was tested in six studies [27–29, 31, 32, 35] with 2971 cases and 3150 controls. Random-effects models were used to calculate the pooled ORs. Our findings demonstrated that there were no significant associations between rs12423791 and any myopia in the allelic model (C vs. G: OR = 0.95, 95% CI: 0.81–1.11, *P* = 0.51), dominant model (CC+CG vs. GG: OR = 0.96, 95% CI = 0.80–1.16, *P* = 0.68), recessive model (CC vs. CG+GG: OR = 0.92, 95% CI = 0.73–1.15, *P* = 0.45 and codominant model (CC vs. GG: OR = 0.93, 95% CI = 0.71–1.22, *P* = 0.61 and CG vs. GG: OR = 0.97, 95% CI = 0.82–1.16, *P* = 0.76) (Fig b in S1 File, Table 2).

Rs5742632 was tested in three studies [28, 29, 33] with 1848 cases and 1630 controls. Fixed -effects models were used to calculate the pooled ORs. Our findings suggested that there were no significant associations for the allelic model (C vs. G: OR = 0.97, 95% CI: 0.88–1.07, *P* = 0.57), dominant model (CC+CG vs. GG: OR = 1.01, 95% CI = 0.88–1.17, *P* = 0.88), recessive model (CC vs. CG+GG: OR = 0.89, 95% CI = 0.75–1.07, *P* = 0.22 and codominant model (CC vs. GG: OR = 0.91, 95% CI = 0.75–1.12, *P* = 0.38 and CG vs. GG: OR = 1.04, 95% CI = 0.90–1.21, *P* = 0.59) (Fig c in S1 File, Table 2).

Rs10860862 was tested in three studies [31, 34, 35] with 1967 cases and 2182 controls. Fixed-effects models were used to calculate the pooled ORs. Our findings demonstrated that there were no significant associations between rs10860862 and any myopia in the allelic model (T vs. G: OR = 1.02, 95% CI: 0.91–1.14, *P* = 0.80), dominant model (TT+TG vs. GG: OR = 1.00, 95% CI = 0.87–1.16, *P* = 0.98), recessivemodel (TT vs. TG+GG: OR = 1.06, 95% CI = 0.84–1.35, *P* = 0.62 and codominant model (TT vs. GG: OR = 1.05, 95% CI = 0.73–1.51, *P* = 0.81 and TG vs. GG: OR = 1.00, 95% CI = 0.86–1.16, *P* = 1.00) (Fig d in S1 File, Table 2).

Rs35766 was tested in three studies [31, 34, 35] with 1964 cases and 2182 controls. Random-effects models were used to calculate the pooled ORs. Our findings suggested that there were no significant associations for the allelic model (G vs. A: OR = 0.93, 95% CI: 0.74–1.16, *P* = 0.51), dominant model (GG+GA vs. AA: OR = 0.95, 95% CI = 0.69–1.31, *P* = 0.77), recessive model (GG vs. GA+AA: OR = 0.81, 95% CI = 0.65–1.00, *P* = 0.05 and codominant model (GG vs. AA: OR = 0.83, 95% CI = 0.56–1.21, *P* = 0.32 and GA vs. AA: OR = 1.01, 95% CI = 0.77–1.32, *P* = 0.97) (Fig e in S1 File, Table 2).

SNP rs5742629 was investigated in three studies [27, 31, 35] with 1169 cases and 1283 controls. Our findings indicated that no significant associations were present between this SNP and any myopia using the allelic model (G vs. A: OR = 0.94, 95% CI: 0.71–1.25, *P* = 0.67), dominant model (GG+GA vs. AA: OR = 1.02, 95% CI = 0.65–1.59, *P* = 0.94), recessive model (GG vs. GA+AA: OR = 0.81, 95% CI = 0.62–1.06, *P* = 0.13 and codominant model (GG vs. AA: OR = 0.87, 95% CI = 0.53–1.42, *P* = 0.58 and GA vs. AA: OR = 1.09, 95% CI = 0.73–1.65, *P* = 0.67) (Fig f in S1 File, Table 2).

### Publication bias

The shape of the funnel plot did not suggest any obvious asymmetry between the seven SNPs and any myopia (see S2 File).

## Discussion

As of August 4, 2021, the Online Mendelian Inheritance in Man (OMIM) database has listed 483 genetic factors associated with myopia. Additionally, two independent genome-wide association studies that involved large cohorts of refractive error patients identified loci at chromosome 15q14 and 15q25 [37, 38]. However, investigating the genetics of complex disorders such as any myopia remains a great challenge. Furthermore, the CREAM consortium conducted multi-center GWAS meta-analyses and identified susceptibility genes that affected diverse biological pathways [39], although they found no evidence of associations between *IGF1* SNPs and myopia. Extended axial length is known to be an important characteristic of the progress of myopia, which is associated with scleral remodeling. It is important to carefully analyze genes in the scleral remodeling pathway. As mentioned above, *IGF1* could contribute to ocular enlargement by changing the structure of the sclera [23].

SNP rs2162679 of *IGF1* has been reported to be associated with several kinds of cancer [40–42], which reminds us that *IGF1* SNPs might play similar role in the onset or progesssion of myopia and cancer. In this study, our meta-analysis shows there is association between *IGF1* rs2162679 and any myopia in codominant model (GA vs. AA) and dominant model (GG+GA vs. AA). The genotype GA and GG+GA in rs2162679 have a lower risk of any myopia than those with the genotype AA. The G allele in this position may protect against the onset or progesssion of myopia.

Rs6214 is located within the intron of *IGF1*. In 2010, Metlapally et al. [43] and Zidan et al. [33] found that rs6214 was positively associated with any myopia/high-grade myopia after correcting for multiple testing. However, in other studies, no significant association for rs6214 was found using single marker analysis [27–32, 34, 35]. Zhuang et al. [31] and Zhao et al. [35] reported that rs12423791 was significantly associated with high myopia in a Chinese population. Although Mak et al. [29] found no association in a Chinese population, they identified a three-SNP haplotype consisting of rs12423791 with a significant association between high myopia and control participants using a variable-sized sliding-window strategy. The final results of this meta-analysis indicated that rs6214 and rs12423791 were not associated with any myopia. In this present study, we included three studies for meta-analysis of rs5742632, rs5742632, rs35766 and rs5742629 respectively. However, our analysis revealed no association between these SNPs and any myopia in genetic models.

Additionally, some other SNPs are notable, although we could not carry out meta-analysis. For example, rs12579077 and rs35767 were reported in the study of Mak et al. [29] in 2012, which are both located in the promoter region. Additionally, we have conducted SNP function prediction using the “SNPinfo Web Server”, which suggests that the two SNPs may play important roles in susceptibility to high myopia. Additionally, rs12423791, rs7956547 and rs5742632 comprise a unit that may be associated with genetic susceptibility to high myopia in Chinese adults. Rs5742714 is located in the 3ʹ-UTR of the *IGF1* gene. Variants in the 3ʹ-UTR affect the binding region of microRNA, which plays an important role in disease by regulating translation of mRNA. Rs35766 is located in the 5ʹ-near region. The 5ʹ-near region may have a role in regulating the transcription of mRNA. In our present study, we found that rs35766 and rs1457601 were detected by one study [31] that suggested associations with high myopia. Although these two SNPs are located in the 5ʹ-near region of the *IGF1* gene, which may play important roles in the process of transcriptional regulation, these associations need to be validated in further studies. Additionally, rs1457601 also is located in the 5ʹ-near region. ALD map based on 1000 genome data provides potential evidence of a haplotypic effect between SNP rs1457601 and other SNPs, such as rs74633605, rs79196465 and rs79218426. Accordingly, the rs1457601 haplotypes also warrant future study.

There are several limitations to this present study. Firstly, the SNPs that we studied were all located in one chromosome according to existing data and haplotype analysis was not performed, which may have affected our results to some extent. It is necessary to pay more attention to haplotype analysis and SNPs on other chromosomes, especially those located in functional regions. Secondly, the major ethnic subjects was Asian, such as Japanese and Chinese. Besides, there are few studies on the polymorphism of any myopia, especially mild and moderate myopia. This two may affect the extrapolation of the conclusions. It is necessary to conduct further studies in other ethnic populations and subjects with different degrees of myopia. Thirdly, myopia is a complex disease affected by hereditary and environmental factors. Environmental factors may cause genetic changes. Gene-environment interactions should also be taken into consideration.

## Conclusion

In conclusion, this meta-analysis suggests that the G allele of the *IGF1* rs2162679 SNP is a potential protective factor for any myopia, which is worth further researches. Haplotype analysis and gene-environment interactions should also be taken into consideration.

## Supporting information

S1 File Meta-analysis of the association of other *IGF1* SNPs with any myopia. (DOCX)

S2 File Funnel plot analysis for publication bias. (DOCX)

S3 File Search strategy. (DOCX)

S4 File Prisma 2009 checklist. (DOCX)
