# Supplementary material for: The G allele of the IGF1 rs2162679 SNP is a potential protective factor for any myopia: Updated systematic review and meta-analysis
Source: PLoS One. 2022 Jul 21;17(7):e0271809. doi: 10.1371/journal.pone.0271809 (PMC9302841; doi:10.1371/journal.pone.0271809)
Supplement: S1 File — (DOCX) [file pone.0271809.s001.docx]

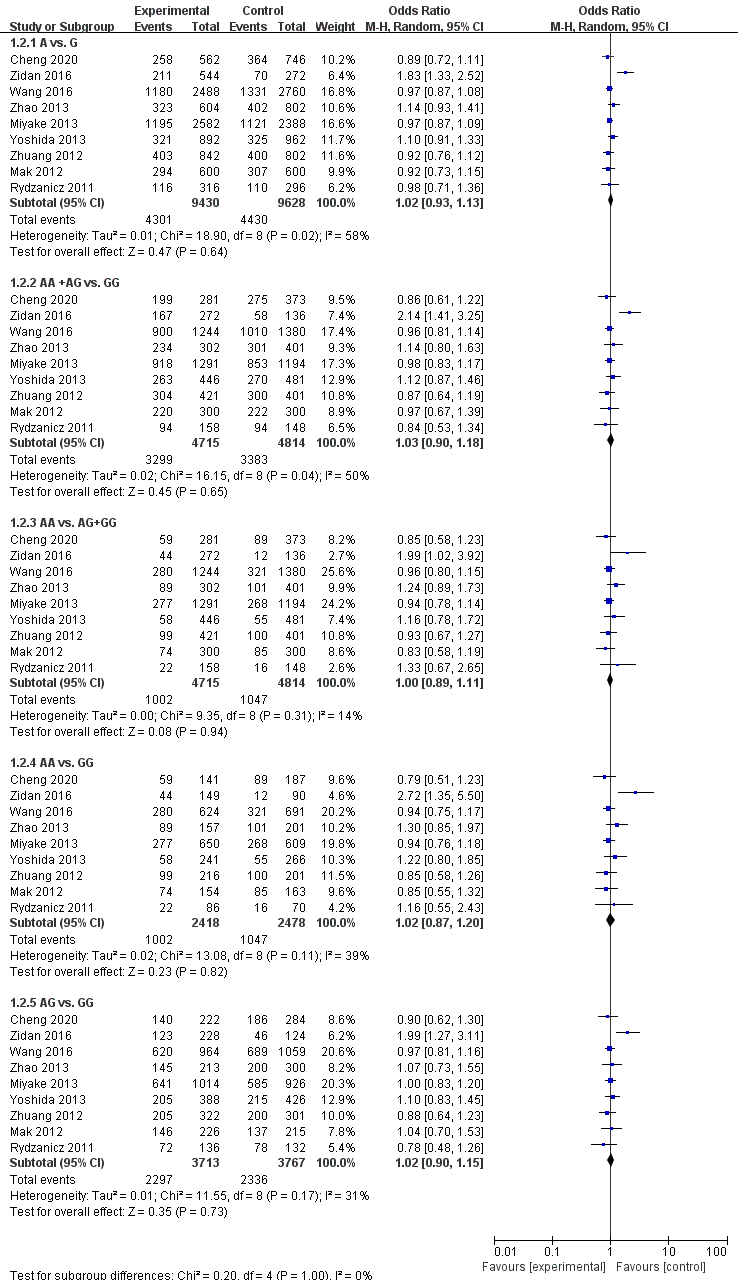


Figure a

Meta-analysis of the association of *IGF1* rs6214 with any myopia.


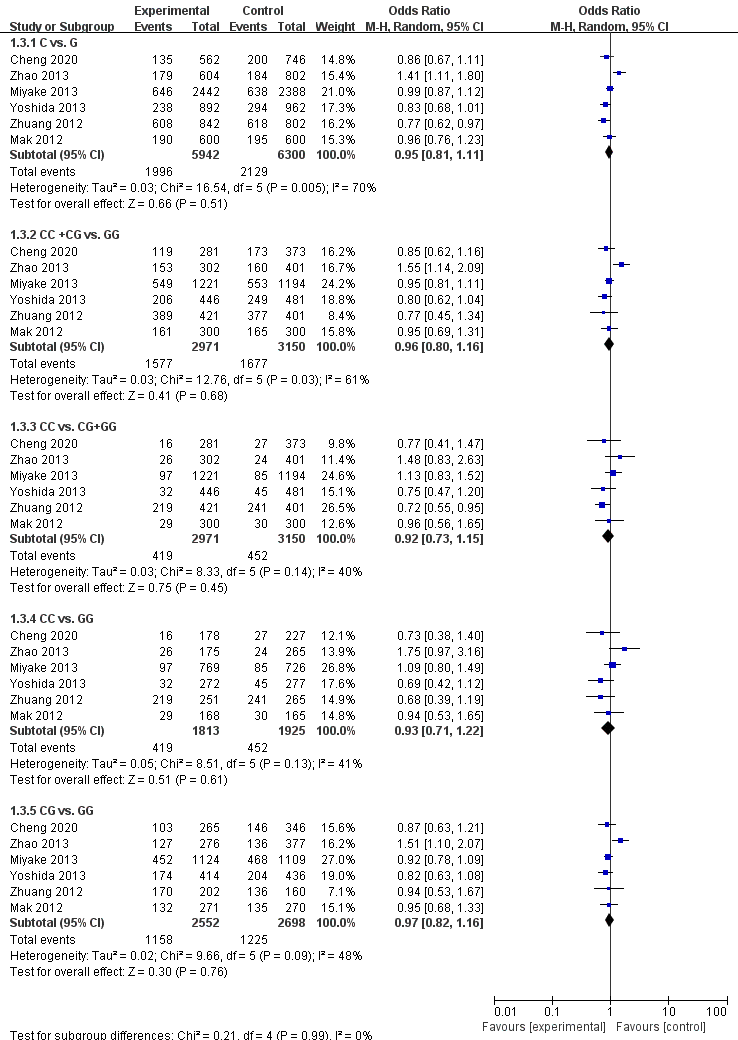


Figure b

Meta-analysis of the association of *IGF1* rs12423791 with any myopia.


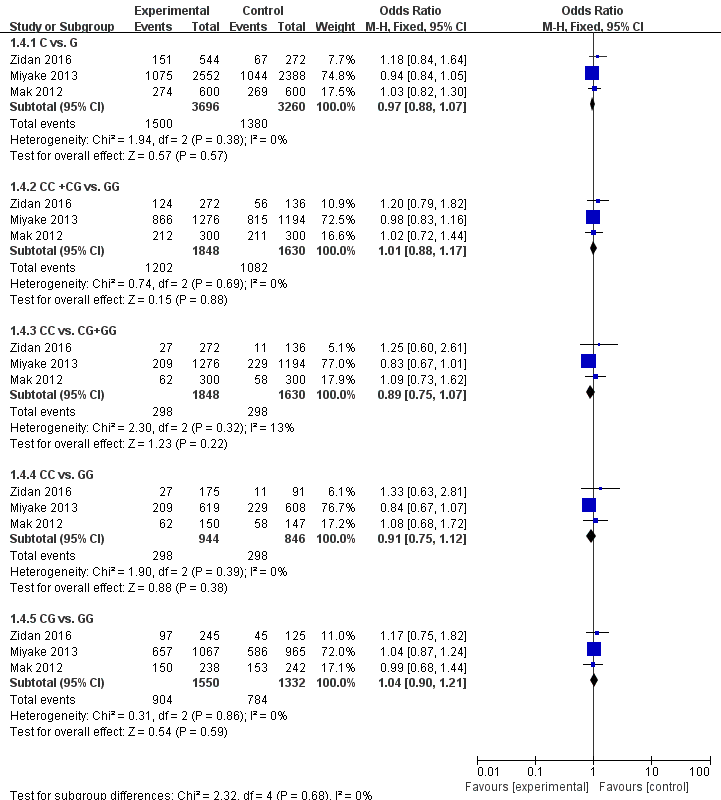


Figure c

Meta-analysis of the association of *IGF1* rs5742632 with any myopia.


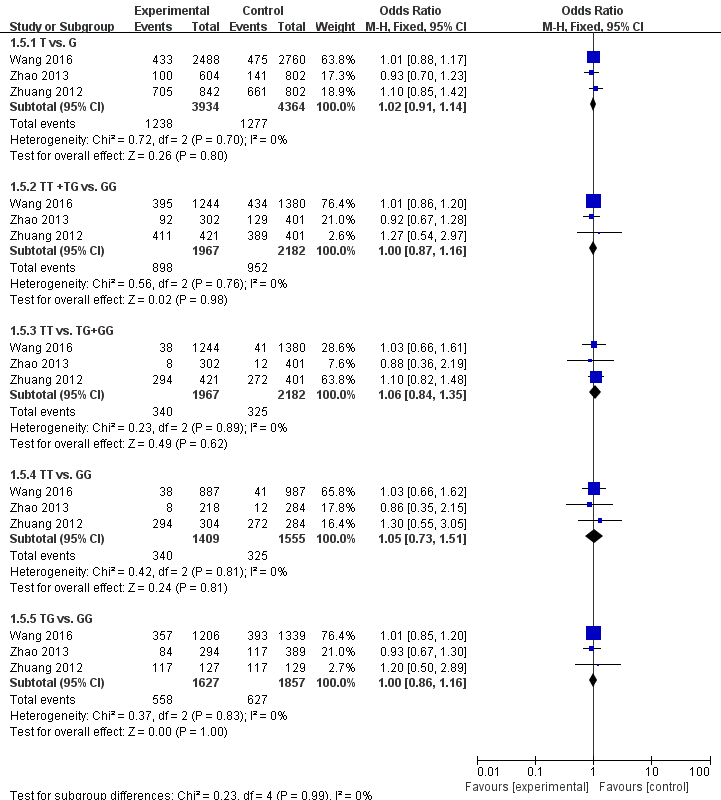


Figure d

Meta-analysis of the association of *IGF1* rs10860862 with any myopia.


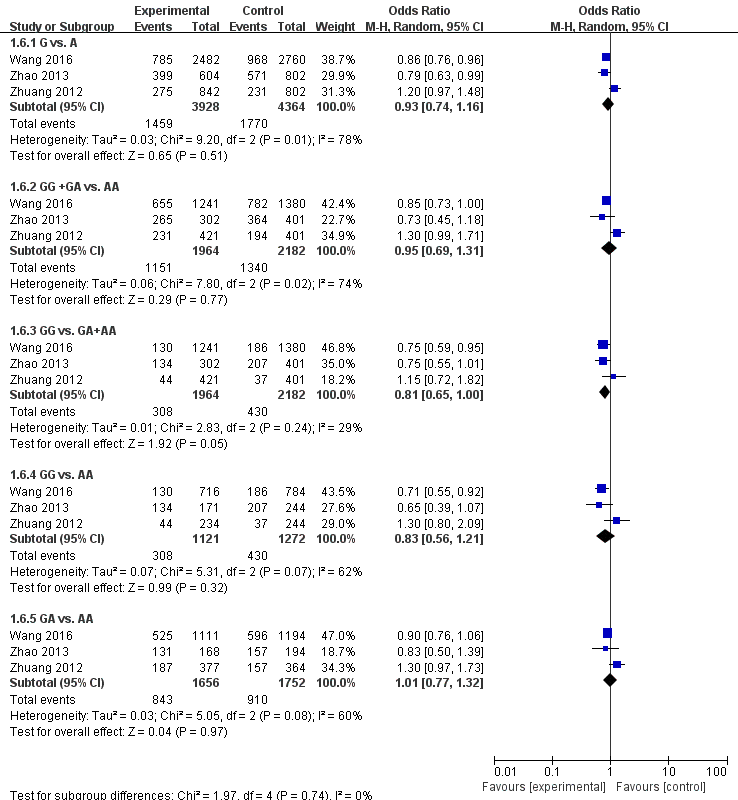


Figure e

Meta-analysis of the association of *IGF1* rs35766 with any myopia.


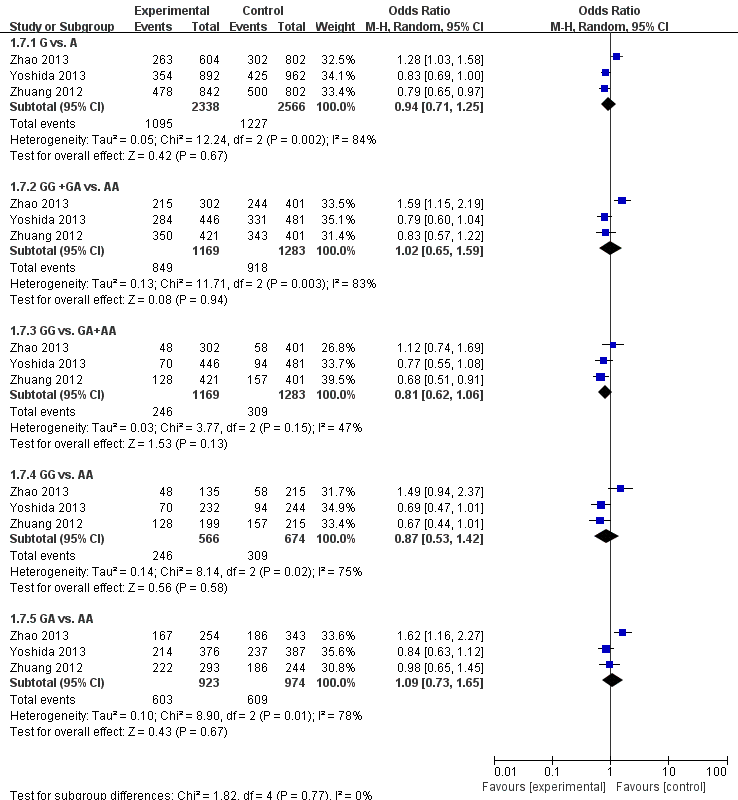


Figure f

Meta-analysis of the association of *IGF1* rs5742629 with any myopia.
