# Supplementary material for: The G allele of the IGF1 rs2162679 SNP is a potential protective factor for any myopia: Updated systematic review and meta-analysis
Source: PLoS One. 2022 Jul 21;17(7):e0271809. doi: 10.1371/journal.pone.0271809 (PMC9302841; doi:10.1371/journal.pone.0271809)
Supplement: S2 File — (DOCX) [file pone.0271809.s002.docx]

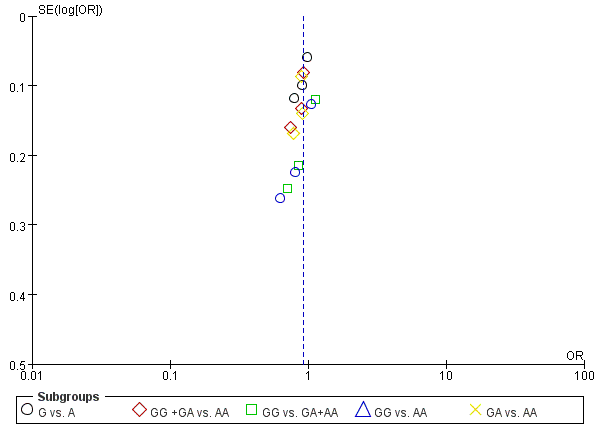


Figure a

Funnel plot analysis for publication bias as to SNP rs2162679.


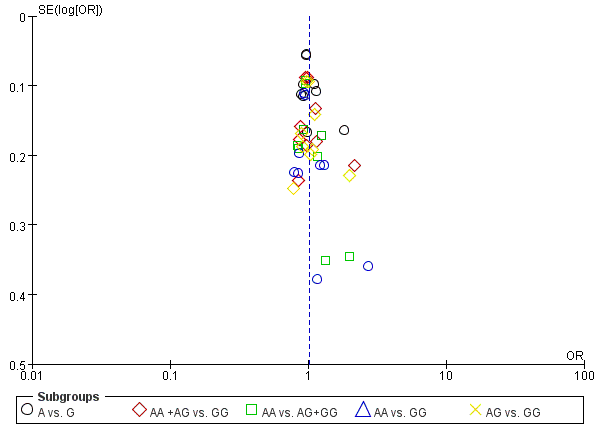


Figure b

Funnel plot analysis for publication bias as to SNP rs6214.


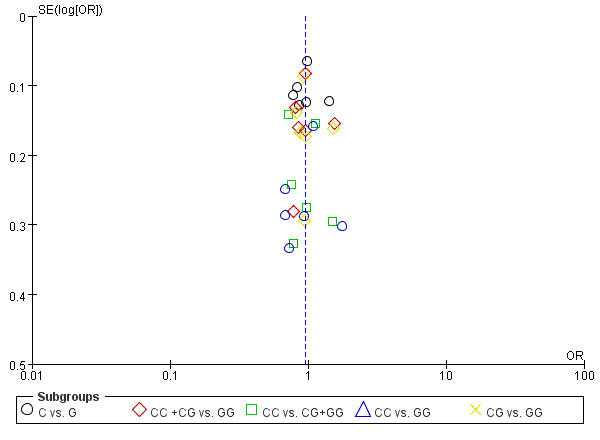


Figure c

Funnel plot analysis for publication bias as to SNP rs12423791.


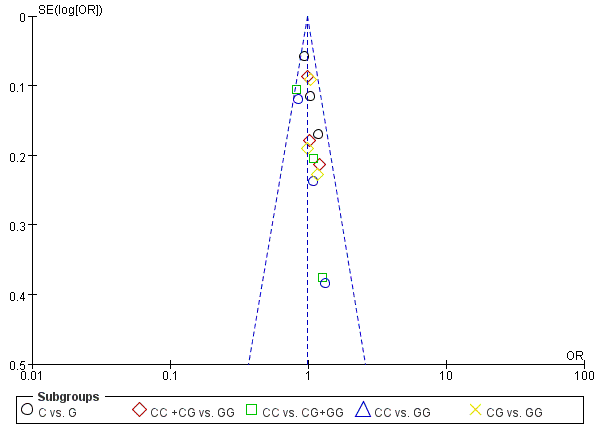


Figure d

Funnel plot analysis for publication bias as to SNP rs5742632.


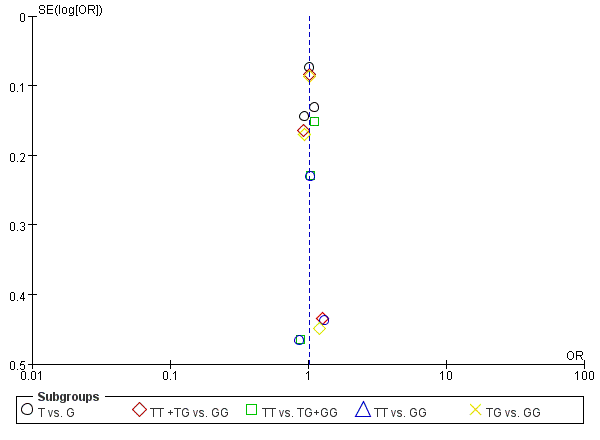


Figure e

Funnel plot analysis for publication bias as to SNP rs10860862.


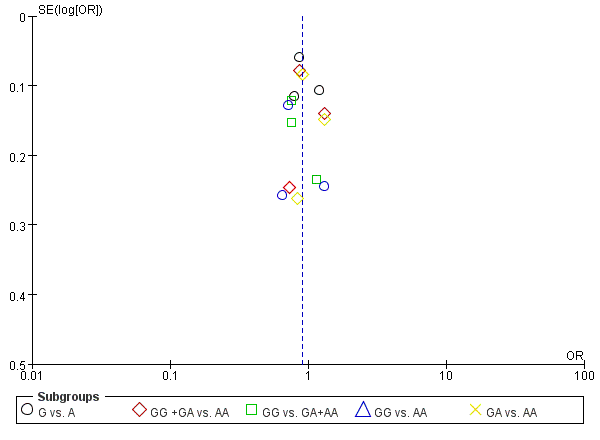


Figure f

Funnel plot analysis for publication bias as to SNP rs35766.


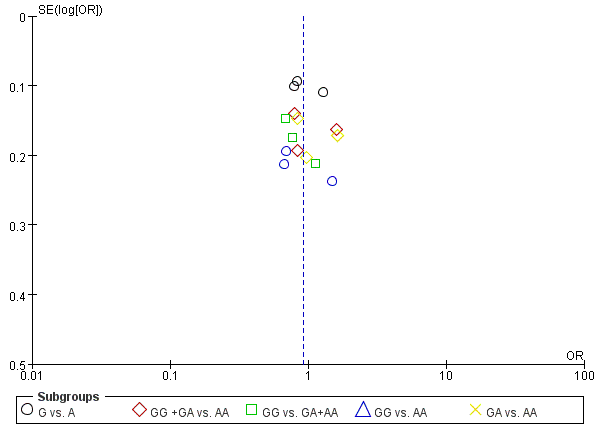


Figure g

Funnel plot analysis for publication bias as to SNP rs5742629.
