# Supplementary material for: The G allele of the IGF1 rs2162679 SNP is a potential protective factor for any myopia: Updated systematic review and meta-analysis
Source: PLoS One. 2022 Jul 21;17(7):e0271809. doi: 10.1371/journal.pone.0271809 (PMC9302841; doi:10.1371/journal.pone.0271809)
Supplement: S3 File — (DOCX) [file pone.0271809.s003.docx]

Search strategy in pubmed.

1=(((IGF-1 or IGF1 or mecasermin or somatomedin C or insulin-like growth factor 1)) AND ((SNP or polymorphism or nucleotide or variant or genome or mutation or (locus or loci) or DNA or exon or exome or intron or allele or gene or haplotype or genotype))) AND ((myopia or nearsighted or near sight or refractive error or shortsighted or short light or myope or myopic defocus or HM))

2=(((IGF-1 or IGF1 or mecasermin* or somatomedin C or insulin-like growth factor 1)) AND ((SNP* or polymorphism* or nucleotide* or variant* or genome* or mutation* or (locus or loci) or DNA or exon* or exome* or intron* or allele* or gene* or haplotype* or genotype*))) AND ((myopia* or nearsighted* or near sight or refractive error or shortsighted* or short light or myope* or myopic defocus or HM))

3=((Myopia[MeSH Terms]) AND (Polymorphism, Single Nucleotide[MeSH Terms])) AND (insulin-like growth factor 1[MeSH Terms])

1 OR 2 OR 3
