# Supplementary material for: The G allele of the IGF1 rs2162679 SNP is a potential protective factor for any myopia: Updated systematic review and meta-analysis
Source: PLoS One. 2022 Jul 21;17(7):e0271809. doi: 10.1371/journal.pone.0271809 (PMC9302841; doi:10.1371/journal.pone.0271809)
Supplement: Attachment — Submitted filename: Response to Reviewers.doc [file pone.0271809.s005.doc]

Thank you for your question. The point-by-point responses to specific comments are as follows:

Journal Requirements:

1. Please ensure that your manuscript meets PLOS ONE's style requirements, including those for file naming.

Response: We have modified the format as requested by PLOS ONE, including those for file naming.

2. Please confirm that you have included all items recommended in the PRISMA checklist including identifying the study as a meta-analysis or systematic review in the title.

Response: We have included items “Updated systematic review and meta-analysis” in the title. And we also updated PRISMA checklist and provided registration information including registration number.

3. Thank you for stating the following financial disclosure:

No

d) If you did not receive any funding for this study, please state: “The authors received no specific funding for this work.”

Please include your amended statements within your cover letter; we will change the online submission form on your behalf.

Response: The statement " The authors received no specific funding for this work." has been included in the cover letter.

4. Thank you for stating the following in your Competing Interests section:

NO authors have competing interests

If you have no competing interests, please state "The authors have declared that no competing interests exist." This information should be included in your cover letter; we will change the online submission form on your behalf.

Response: The statement "The authors have declared that no competing interests exist." has been included in the cover letter.

5. Please ensure that you include a title page within your main document. You should list all authors and all affiliations as per our author instructions and clearly indicate the corresponding author.

Response: We have included a title page within the main document, which includes all authors and all affiliations and clearly indicates the corresponding author.

6. Please amend your manuscript to include your abstract after the title page.

Response: We have included the abstract after the title page.

7. Please include a separate caption for each figure in your manuscript.

Response: The separate caption for each figure in our manuscript has been included.

8. Please ensure that you refer to Figure 2 in your text as, if accepted, production will need this reference to link the reader to the figure.

Response: We have referred to Figure 2 as Fig 2 in our text.

9. Please include your tables as part of your main manuscript and remove the individual files. Response: Please note that supplementary tables (should remain/ be uploaded) as separate "supporting information" files.
Response: We have included Table 1 and Table 2 as part of our main manuscript and removed the individual files. The supplementary tables are uploaded as separate "supporting information" files.

Reviewer #1:

1. in this study the comparisons were done between the different groups and SNPs using analysis of variance, the authors need to do multiple testing to reject false hypotheses.
Response: In meta-analysis, the number of events and total participates were used to recalculate the total OR value and 95%CI of included studies. At present, due to the limitations of meta-analysis software, the results of multiple comparisons cannot be analyzed.

2. In the Results rs2162679, "significant association" should be changed to "suggestive association."
Response: In the Results rs2162679, "significant association" has been changed to "suggestive association."

3. In the Results , the paragraph descriptions of rs2162679 that supporting file should be (Table 1, Table 2) instead of (Fig 1, Table 2), this paragraph not related with Fig 1.
Response: In the Results , the paragraph descriptions of rs2162679 that supporting file have been changed to (Fig 2, Table 2) instead of (Fig 1, Table 2).

4. in Abstract and Discussion the author said rs2162679 was significantly associated with any myopia in the codominant model (GA vs. AA: OR = 0.87, 95% CI: 0.76-1.00) and the dominant model (GG+GA vs. AA: OR = 0.88, 95% CI = 0.78-1.00). but in results the author said rs2162679 "there were no significant associations for the allelic model (G vs. A: OR = 0.93, 95% CI: 0.85-1.02, P=0.14), dominant model (GG+GA vs. AA: OR = 0.88, 95% CI = 0.78-1.00, P=0.05), recessive model (GG vs. GA+AA: OR = 0.99, 95% CI = 0.82-1.19, P=0.92 and codominant model (GG vs. AA: OR = 0.92, 95% CI = 0.76-1.13, P=0.43).", this is inconsistency, authors need to correct that.
Response: Codominant model contains two types, that is (GA vs. AA) and (GG vs. AA). In our study, codominant model (GA vs. AA: OR = 0.87, 95% CI: 0.76-1.00) was suggestively associated with any myopia, while there was no significant association for the other codominant model (GG vs. AA: OR = 0.92, 95% CI = 0.76-1.13, P=0.43). So this is not inconsistency.

5. the authors need add in figure legend for Fig 1 and Fig 2 .

Response: Figure legends for Fig 1 and Fig 2 have been added.

Reviewer #2:

1. Please include “meta-analysis” in the title

Response: We have included “Updated systematic review and meta-analysis” in the title.

2. Add a reference to Page 7 Line 12-13 for the “Myopia has already become the second most common cause of legal blindness”

Response: Two references have been added for the “Myopia has already become the second most common cause of legal blindness”.

3. Page 8 Line 9: delete “[22]”

Response: We have deleted “[22]” in this place.

4. Table 1 is truncated on the right side and is not fully shown in the manuscript

Response: The table 1 has been adjusted and it is fully shown in the manuscript.

5. Check the format of all references.

Response: We have checked the format of all references as requested by PLOS ONE.
